# Enhanced molecular release from elderly bone samples using collagenase I: insights into fatty acid metabolism alterations

**DOI:** 10.1186/s12967-024-04948-8

**Published:** 2024-02-09

**Authors:** Amir Mohammad Malvandi, Esra Halilaj, Martina Faraldi, Laura Mangiavini, Simone Cristoni, Valerio Leoni, Giovanni Lombardi

**Affiliations:** 1 Laboratory of Experimental Biochemistry & Molecular Biology, IRCCS Istituto Ortopedico Galeazzi, Via Cristina Belgioioso 173, 20157 Milan, Italy; 2 School of Biosciences and Veterinary Medicine, University of Camerino, Camerino, Italy; 3 Department of Biomedical Sciences for Health, University of Milan, Milan, Italy; 4 ISB-Ion Source & Biotechnologies Srl, Bresso, Italy; 5 Department of Laboratory Medicine, University of Milano-Bicocca, Azienda Socio Sanitaria Territoriale Della Brianza, ASST-Brianza, Desio Hospital, Desio, Italy; 6 Department of Athletics, Strength and Conditioning, Poznań University of Physical Education, Poznań, Poland

**Keywords:** Bone metabolism, Elderly, Aging, Metabolic pathways, Metabolomics

## Abstract

**Background:**

Bone is a metabolically active tissue containing different cell types acting as endocrine targets and effectors. Further, bone is a dynamic depot for calcium, phosphorous and other essential minerals. The tissue matrix is subjected to a constant turnover in response to mechanical/endocrine stimuli. Bone turnover demands high energy levels, making fatty acids a crucial source for the bone cells. However, the current understanding of bone cell metabolism is poor. This is partly due to bone matrix complexity and difficulty in small molecules extraction from bone samples. This study aimed to evaluate the effect of metabolite sequestering from a protein-dominated matrix to increase the quality and amount of metabolomics data in discovering small molecule patterns in pathological conditions.

**Methods:**

Human bone samples were collected from 65 to 85 years old (the elderly age span) patients who underwent hip replacement surgery. Separated cortical and trabecular bone powders were treated with decalcifying, enzymatic (collagenase I and proteinase K) and solvent-based metabolite extraction protocols. The extracted mixtures were analyzed with the high-resolution mass spectrometry (HRMS). Data analysis was performed with XCMS and MetaboAnalystR packages.

**Results:**

Fast enzymatic treatment of bone samples before solvent addition led to a significantly higher yield of metabolite extraction. Collagenase I and proteinase K rapid digestion showed more effectiveness in cortical and trabecular bone samples, with a significantly higher rate (2.2 folds) for collagenase I. Further analysis showed significant enrichment in pathways like de novo fatty acid biosynthesis, glycosphingolipid metabolism and fatty acid oxidation-peroxisome.

**Conclusion:**

This work presents a novel approach for bone sample preparation for HRMS metabolomics. The disruption of bone matrix conformation at the molecular level helps the molecular release into the extracting solvent and, therefore, can lead to higher quality results and trustable biomarker discovery. Our results showed β-oxidation alteration in the aged bone sample. Future work covering more patients is worthy to identify the effective therapeutics to achieve healthy aging.

**Supplementary Information:**

The online version contains supplementary material available at 10.1186/s12967-024-04948-8.

## Introduction

Bone turnover is a continuous mechanism essential for bone integrity in response to the mechanical or physiochemical needs of the body [1]. The energy required for this dynamics correlates bone metabolism with general body homeostasis and the prevention of fractures [2]. In part, elderly frailty comes from a loss of bone tissue integrity that imposes a higher risk of fracture and disability and less supply of minerals to the body [3]. Macroscopically, bone is made up by two structural portions: the dense (cortical) and the sponge-like (trabecular). The osteon is the fundamental structural unit of cortical bone and consists of concentric layers of mineralized bone tissue surrounding a central canal. Each osteon contains osteocytes, osteoblasts, and osteoclasts, which work harmoniously to maintain bone homeostasis. Osteocytes are the most abundant cells within the osteon and are responsible for sensing mechanical stresses and orchestrating bone remodeling accordingly. Osteon, as the functional unit of cortical bone, is physiologically active, has direct contact with the bloodstream, and is subjected to constant remodeling. Trabecular bone is distinguished by its lattice-like structure, comprised of a network of thin, interconnected plates and struts. This design provides strength and lightness to bones, making them well-suited for their weight-bearing functions. Trabecular bone is found primarily in the epiphyses of long bones, the core of vertebrae, and within the structure of flat bones. The spaces within the trabecular structure are filled with bone marrow, which plays a central role in hematopoiesis and contributes to the body’s immune system. Understanding trabecular bone metabolism is also closely tied to studying bone remodeling units (BRUs). BRUs consist of osteoclasts and osteoblasts working together as a functional unit to resorb and replace bone tissue. Molecular metabolomics has enabled the identification of specific proteins, enzymes, and factors mediating the communication between these cells and the signaling pathways that dictate bone remodeling. However, the knowledge regarding bone metabolomics is currently limited by the difficulty in metabolite extraction. The mineralized long-chain protein structures have a high potential to trap biomolecules that fail the traditional phase extraction methods to extract small molecules [4].

In aging, metabolic pathways within bone cells undergo significant alterations, particularly in the molecular interactions governing osteoblast and osteoclast activities [5]. The dysregulation of pivotal signaling pathways such as Wnt and Notch contributes to impaired bone formation and increased resorption [6]. The decline in insulin-like growth factor-1 (IGF-1) signaling hinders osteoblast function [7]. Overexpression of receptor activator of nuclear factor-κB ligand (RANKL) and reduced osteoprotegerin (OPG) expression disrupts the balance between osteoclastogenesis and inhibition, leading to enhanced bone resorption in the elderly [8]. Age-related autophagy and mitochondrial function changes compromise cellular homeostasis and induce oxidative stress that triggers inflammatory responses, further promoting osteoclastogenesis [9]. This complex interplay shows a possible shift in the metabolic pathways, which can then orchestrate the cell physiology with metabolites/energy availability. However, the current knowledge is limited. A clearer vision is essential for comprehensive understanding to define effective strategies to promote bone health in elevated ages, enhancing our ability to prevent and manage age-related skeletal disorders.

Currently the knowledge is compromised by the difficulties in metabolites extraction form the samples [10]. Bone’s inherent heterogeneity, encompassing different cell types and regions, makes it challenging to obtain representative samples. Moreover, the mineralized matrix of bone can impede metabolite accessibility, potentially skewing results. Extracting metabolites from bone is complicated by low metabolite abundance, hindering comprehensive profiling [11]. Demineralization processes are necessary but can introduce biases and degradation risks. Contaminants from neighboring tissues and fluids may infiltrate the sample. Additionally, limited sample sizes and metabolites’ sensitivity to environmental factors demand meticulous extraction protocol optimization. The choice of extraction solvents plays a pivotal role, as metabolite solubility differs. Metabolite identification in bone samples is complex, and accurate quantification is challenging due to low abundance and potential matrix effects [12]. Overcoming these issues is vital for advancing our comprehension of bone metabolism and its implications for osteoporosis and metabolic bone diseases. In this work, we aimed to refine extraction and analytical methods to enhance the reliability and accuracy of bone metabolite profiling.

## Methods

### Human sample collection

Three pools of cortical and trabecular bone powders were used from the bone samples obtained from 10 patients (males and females) aged 65 to 85 who underwent elective hip replacement surgery at IRCCS Istituto Ortopedico Galeazzi, Milan, Italy. The age span of patients was defined according to the World Health Organization (WHO) definition of elderly age [13]. Selected patients were not treated with drugs that can affect bone or glucose metabolism. The participants with diseases known to affect bone metabolism, alcohol and tobacco consumers, and bone tumors or other diseases involving surgery sites other than osteoarthritis were excluded. Bone specimens were processed as described previously [14] and powders from cortical and trabecular parts were kept at – 80 ℃ for further analysis.

### Metabolites extraction

In this work, we applied four modifications to the general condition of tert-butyl methyl ether (MTBE) based biphasic extraction protocol, called Matyash [15] as follows: addition of enzymatic digestion, (1st) collagenase I (Worthington, USA) due to dominancy of collagen presence in the bone matrix; and (2nd) proteinase K (Invitrogen, life technologies Italy) since it is a widely accepted proteinase to increase metabolite extraction yield on other biological matrices; EDTA mediated decalcification (3rd) to explore the effect of calcium on metabolites sequestration; stepwise addition (4th) of solvent phase with the same volumetric ratio as original Matyash; and the MTBE original extraction protocol (5th).

**Fig. 1:**
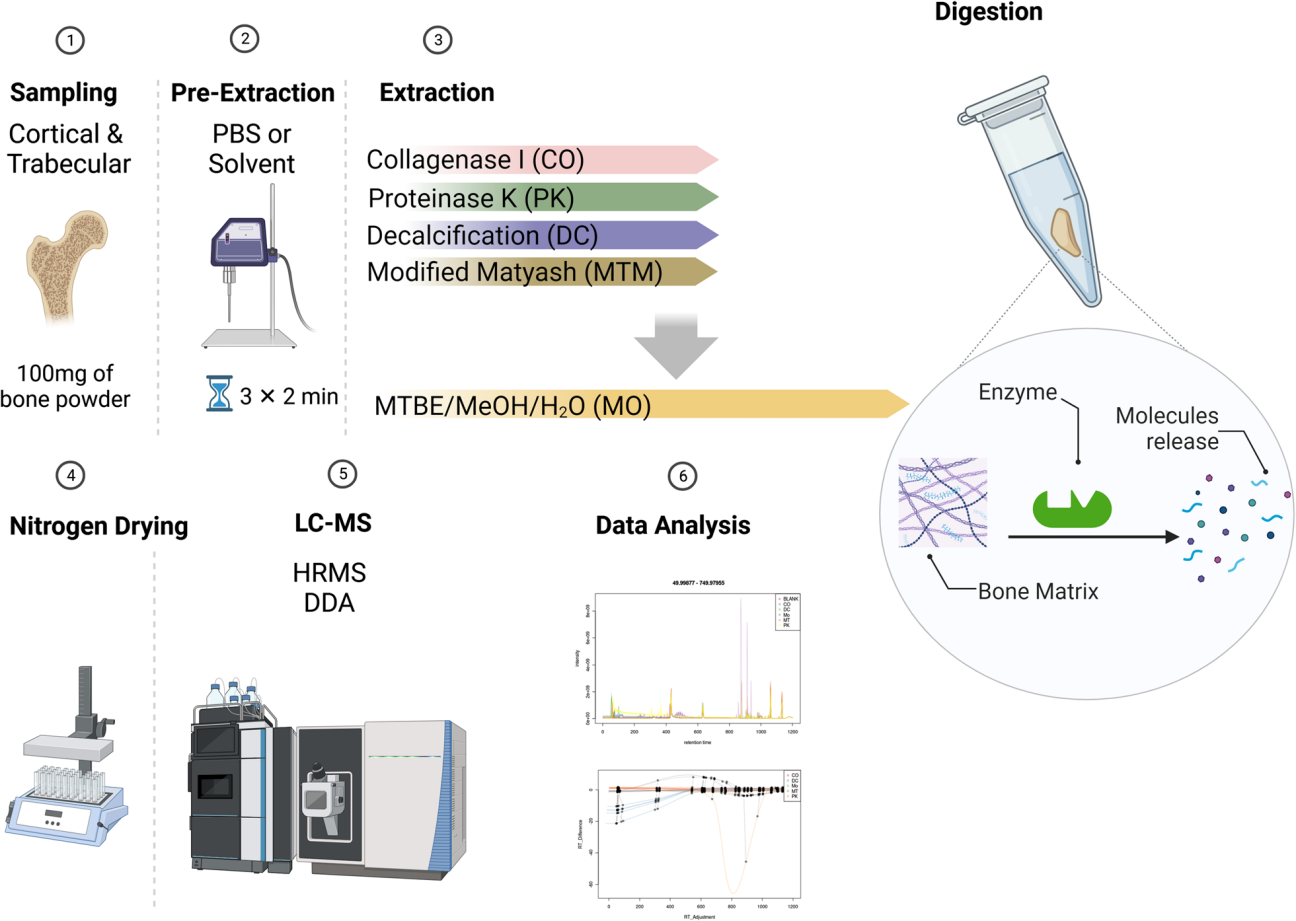
The experimental design, grouping and steps (left part). Schematic presentation (right panel) of enzymatic digestion effect increasing molecular release from the bone matrix

Bone powders were weighed at cold and 25 falcon tubes containing 100 µg of bone powder each were prepared for 5 different metabolite isolation conditions (Fig. 1). For the collagenase I and proteinase K groups, samples were resuspended in sterile filtered PBS (made with LC grade water from Merck, Italy). The decalcifying (DC) group was resuspended in EDTA 20% in LC Grade water from Merck, Italy). The modified Matyash (MTM) group, powders were resuspended in MTBE (HPLC Plus from Merck, Italy) and the Matyash original group samples were resuspended in MTBE/methanol solution. All samples were sonicated for three cycles of 2 min at 65% potency using a horn sonicator (Vibra cell^™^, sonics & materials, inc. USA). The enzymatic digestion was performed for 15 min at 50 ℃ for collagenase I and proteinase K groups’ samples by the addition of 100 µL of collagenase I (1 mg/mL) or proteinase K (20 mg/mL) to each corresponding sample. The samples were then topped up to 12 mL with the MTBE/MeOH/H_2_O in a volumetric ratio of 2.1:2.1:0.8 (v/v/v). The samples were then sonicated for 30 min in an ultrasonic water bath. After 24 h incubation at – 20 ℃, the samples were dried with N_2_ gas fellow (Col-Parmer, UK), resuspended in 100 µL of MeOH (70% in H2O), and kept at – 80 ℃ until the analysis. A flowchart design of different steps is provided in Additional file 1: Fig. S2.

### Untargeted discovery metabolomics

The samples were analyzed with an ultra-performance liquid chromatography UPLC coupled with a high-resolution mass spectrometer (Vanquish UPLC, Orbitrap Exploris^™^ 120 Mass Spectrometer, Thermofisher). The analysis course was set for 30 min with a gradient of MeOH (with 0.1% of Formic Acid) from 2 to 80% against H2O (with 0.1% of Formic Acid) using a C18 LC column 100 × 3.0 mm (Kinetex^®^ 2.6 µm Polar C18 100 Å, Phenomenex Inc. Torrance, CA USA). The analysis was performed with the data-dependent analysis (DDA) approach, and both scans were set at high-resolution conditions (MS1 at 120000, MS2 at 15000 FWHM). Raw Data was analyzed using the XCMS package (v.3) at the R software platform (v. 4.3.0) [16].

The absolute values (the total aligned features extracted from each method) were used to run Chi-Square tests on the R software platform (v. 4.3.0) to find statistical significance for the yield of each method used in this study. The test was done for all the methods together and every two matches comparisons (Additional file 1: Table S3 summarizes all data). The p-value less than 0.05 is considered significant.

### Artificial intelligence based Quality control (AI-QC)

Artificial intelligence (AI) based on the bioinformatics SANIST core has been used to classify the samples [17, 18]. A vectorial database was built using nine spectra extracted by the analyzed groups. The m/z signals were converted to angular vectors and compared with those of unknown samples using the Stein and Scott algorithm [19]. The direct match score and the identification probability percent were used to classify the samples. A direct score match higher than 800 with a probability percent higher than 90% was used to identify the sample.

Additional file 1 Figure S1 shows the data elaboration pipeline. The LC–MS analyses were aligned vs the relative blank. The m/z signals present only in the sample are inserted in the SANIST database and converted into vectors. A set of unknown samples is classified in vectorial similarity with those in the database and following the Stein and Scott approach.

Table 1 reports the results achieved for each classified sample regarding positive or negative recognition, direct match, and probability percentage. All the samples were correctly classified and the replicates with a set quality and recovery rate of more than 85% were used for further analysis.

**Table 1:** Output of SNIST AI-QC pipeline for the selected samples

Name	Avarage direct match	Avarage identity %
Sample 1 series (CO)	876	91
Sample 2 series (DC)	865	92
Sample 3 series (MO)	887	93
Sample 4 series (MT)	889	92
Sample 5 series (PK)	921	91

### Metabolite identifications

The features were statistically analyzed and the significant ones were identified using a locally constructed analytical library within NIST software as described previously [20, 21]. Furthermore, spectral analysis and annotation with metabolic pathway enrichment analysis were done using the MetaboAnalystR package and its web-based platform [22]. The hit molecules were cross-checked and validated with both HMDB (11/09/2023 released version under metaboloanlyst platform v.5) according to exact mass and at MS2 levels with the local library (Additional file 1: Figure S3).

We applied multiple statistical evaluations to identify the significant metabolites from global profiling analysis. First, the dataset was normalized based on the interquantile range and transformed using natural logarithm. The scaled dataset based on mean-centered and divided by the standard deviation of each variable was used for further evaluations. K-means PCA and hierarchical clustering were used to find the most different groups of data in the whole data set and then with the volcano plot, the significant single features were extracted. We proceeded the data interpolation with Enrichment analysis for both signaling pathways and the chemical type of analyzed metabolites.

## Results

To achieve a higher level of metabolite recognition, we introduced four modifications to the MTBE-based metabolite extraction protocol reported by Sostare et al. [15].

**Fig. 2:**
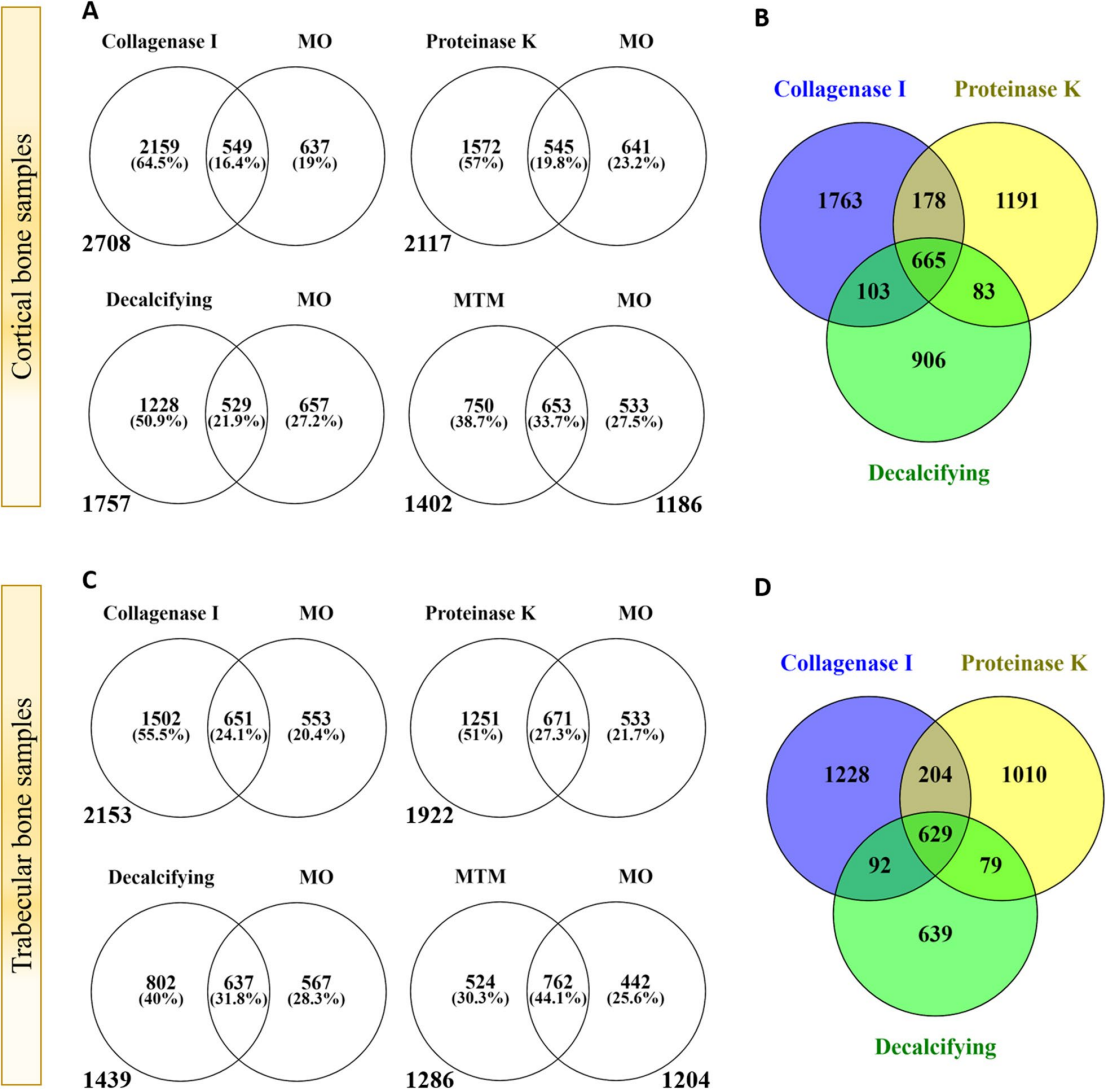
Venn diagram comparison of the aligned features obtained by XCMS analysis. The numbers down to circles are the total number of identified features in the groups. The same blank samples for all the groups has been considered in the analysis. **A** and **B** are the results related to cortical samples; **C** and **D** present the data of the trabecular samples. To make the Venn diagrams, we used the venny 2.1 web application (https://bioinfogp.cnb.csic.es/tools/venny/index.html)

We evaluated if the sequence of solvent addition can help dissolve more small molecules in the MeOH by adding first MTBE and then MeOH to the samples (MTM group). Our results showed the working of this hypothesis significantly in cortical (*p* < 0.0001; Fig. 2A) and not significant in trabecular samples (*p* = 0.1; Fig. 2C), in comparison with the not modified protocol (MO), resulted in a higher number of metabolite extraction and recognition with HRMS. Decalcification significantly enhanced the molecule extraction in cortical (*p* < 0.0001; Fig. 2A) and trabecular (*p* < 0.0001; Fig. 2C) samples and more than the MTM group (*p* = 0.003). Proteinase K treatment significantly increased metabolite extraction yield in trabecular and cortical samples (Fig. 2A and C), which was higher than in DC, MTM (*p* < 0,0001), and MO groups (*p* < 0.0001). The collagenase I treated group yielded more than all other experimental groups (Fig. 2).

We used the m/z mean, followed by the corrected retention time from aligned features obtained by XCMS package results, to compare the yield between the groups. MTM and MO showed a higher similarity (33.7% in cortical and 44.1% in trabecular samples). The recognized features from all groups showed approximately the same similarity ratio between each other, from cortical to trabecular samples (Fig. 2).

**Fig. 3:**
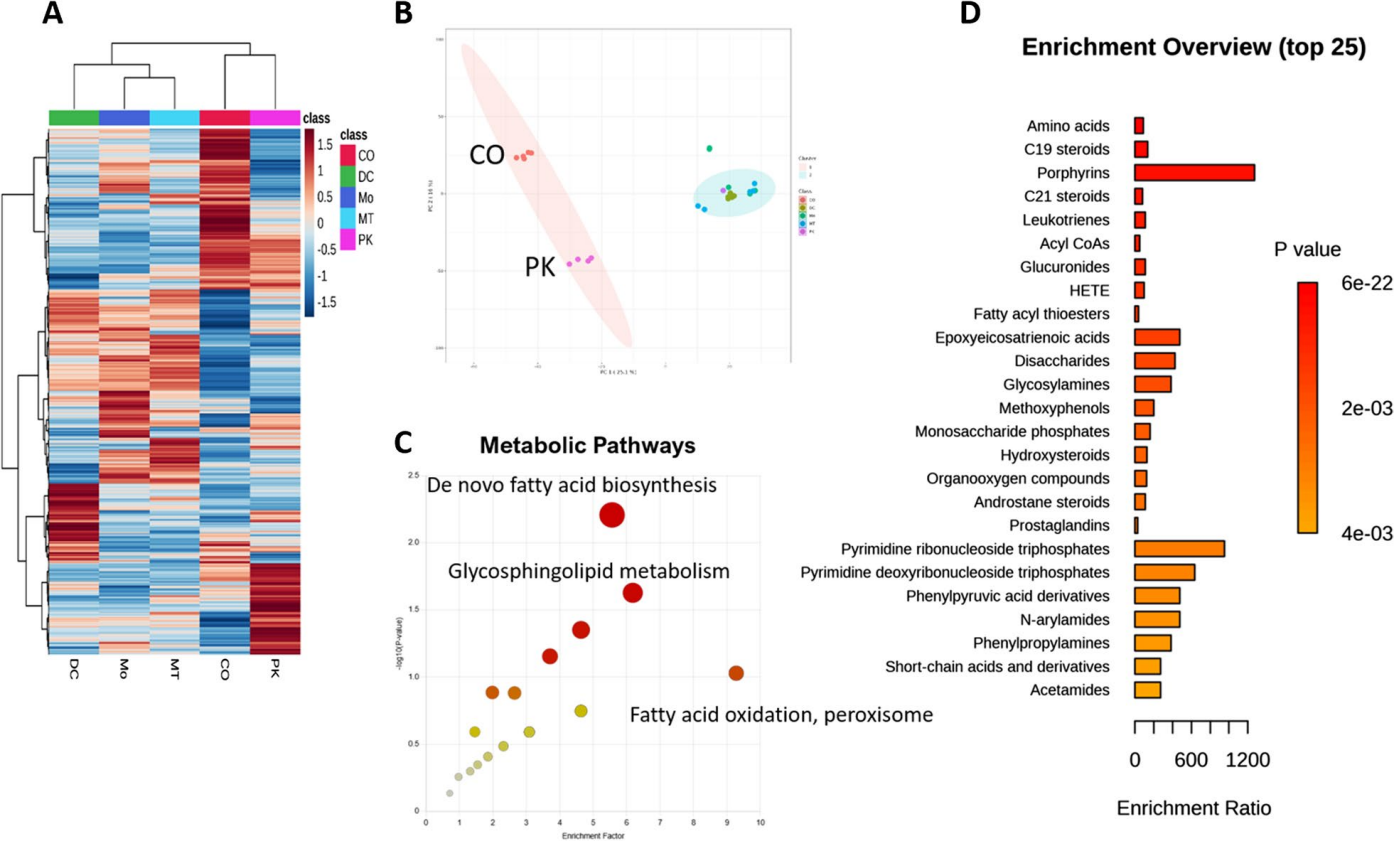
Metabolite identification and analysis from the bone samples. **A** shows heatmap presentation of clustered (Euclidean) of the normalized data (mean per group). K-means PCA analysis (**B**) confirmed the cluster analysis with the distances between PK, CO, and other groups. Functional Enrichment (output of MetaboanalyteR) pathways demonstrated altered pathways with the hit targets from the identified metabolites (**C**). Enrichment analysis for the chemical type of confirmed identified metabolites (based on NIST local library similarity evaluation) covers polar and lipophilic molecules (**D**)

Metabolite identification was performed according to similarity (ppm = 5, all adducts ions, positive mode) to the HMDB database of the derived features’ exact mass and retention time. The discovered hits were confirmed at the MS2 level by cross-database check using a locally developed experimentally validated library in the NIST software platform. The relative expression level of metabolites in the collagenase I group appeared to be higher than that of other groups (Fig. 3A). PCA analysis confirmed the clustering analysis results in showing more similarities between proteinase K and collagenase I groups concerning the others (Fig. 3A and B). Further, K-means PCA analysis showed significant differences between collagenase I- proteinase K and DC-MTM-MO groups (Fig. 3B). Enrichment analysis on the identified metabolites showed more alterations in de novo fatty acid biosynthesis (*P*_*fisher*_ = 0.006215) and glycosphingolipid metabolism (*P*_*fisher*_ = 0.023627), carnitine shuttle (*P*_*fishe*r_ = 0.044611) and purine metabolism (*P*_*fishe*r_ = 0.044611) (Fig. 3C); the complete list of enriched pathways are reported in Additional file 1: Table S1. The extracted metabolites were combined from different polar, non-polar chemical types, lipids and amides (Fig. 3D, Additional file 1: Table S2).

## Discussion

The dense and mineralized bone matrix challenges metabolite extraction for metabolomics studies [23]. It restricts metabolite accessibility, leading to incomplete extraction and reduced recovery [24]. Matrix effects caused by the mineral content can compromise the quantification accuracy in mass spectrometry-based analyses [25]. Metabolite trapping within the matrix and sample heterogeneity further hinder extraction [26]. To enhance the quality of the extracted metabolites, we employed demineralization, enzymatic digestion optimized extraction protocols, and standardization to mitigate variability in results.

Sequentially adding solvent (MTM) with the same v/v ratio helped increase the extraction of metabolites (Fig. 2A). Previous works showed that the ratio or experimental protocol modification can improve the yield [15]. In this work, we tried the sequential addition of solvents. The MTBE dissolves more lipid-type metabolites; therefore, the MeOH phase can dissolve more aqueous phase substances, leading to more metabolite extraction from the bone samples. Considering the m/z mean of the features with the retention time, we have observed a higher similarity between the MTM and MO groups (44.1% in trabecular and 33.7% in cortical samples) in comparison with the other groups with the MO. Therefore, MTM can be considered beneficial only on trabecular samples (18% yield increase).

Decalcification of bone samples led to a significant increase in metabolite extraction (Fig. 2A and C). This observation showed the effect of mineralization on metabolites trapped in the bone matrix. However, the yield of this modification was less than that of enzymatic digestion groups. It showed that in the bone matrix, the presence of long-chain proteins is a dominant effector. Our observation of the groups treated with a short enzymatic digestion confirmed this. The dense mineralized structure of bone can sequester metabolites, rendering them less accessible through traditional extraction methods. Enzymes effectively degrade proteins and other macromolecules, liberating metabolites for subsequent analysis. We adopted proteinase K digestion as a widely used approach to help release the metabolites from the matrix. Proteinase K is a serine protease that can digest a broad range of proteins; therefore, changes in matrix proteins’ conformation allow the release of the trapped small molecules. Although proteinase K digestion has been reported as a practical approach to increase the quality of discovery metabolomics in different types of samples [27, 28], it is the first work applied to the bone sample. The ratio of improvement in metabolite recognition observed in cortical samples was higher than in trabecular ones. It might be due to more protein in the cortical samples. Because the main component of bone matrix is the collagen protein, we further examined whether collagenase I can improve metabolite extraction and identification. We observed a significant increase in the number of extracted/identified metabolites (Fig. 2A, C) and the level of metabolites aligned with all other groups (Fig. 3A). Collagenase I is specific for collagen and particularly useful for bone-related studies where preserving non-collagenous components, such as metabolites, is crucial. In contrast, proteinase K has a broader spectrum of activity and is more commonly used for general protein digestion and DNA/RNA extraction but may not be as selective when it comes to preserving metabolites or specific to bone tissue. Collagen has a distinct repetitive amino acid sequence in its primary structure. Collagenase I is specifically adapted to recognize and cleave the peptide bonds within this structure, making it highly selective for collagen over other proteins in the bone matrix. Collagenase I’s action has a reduced impact on other proteins and metabolites in the sample, which is crucial for preserving the integrity of metabolites, which are often sensitive to enzymatic degradation.

The similarity between groups was similar in both cortical and trabecular samples (Fig. 2B and D). This showed the reproducibility of the analytical procedure and also proved the effectiveness of our quality control pipeline. In this work, we used more than a recovery rate of unique internal controls, an in-house developed AI-based quality control of the analysis. In this way, we have provided more than biological controls; the AI algorithm selected from at least three technical replicates (at the exact analytical parameters). Vectorial comparison of different analytical parameters allowed us to reduce sample variability and keep the best condition for discovery metabolomics. It is critical in *discovery metabolomics* reproducibility and standardization. Traditionally, quality evaluation of untargeted small molecule profiling using LCMS is considered complex and often minimized to a single issue, such as the recovery rate of a few noted external molecules or pooling samples [29]. In comparison, our developed SNIST AI-QC pipeline creates average direct match and average identity percentage as two quantifiable parameters for the quality of a sample. SNIST AI-QC pipeline evaluations consider external standards’ recovery rate, the correlation of analyzed features from different biological/technical replicates, and direct matches between the aligned vectors from different samples. On top of these, the evaluation criteria adapt to the query samples’ condition thanks to the machine learning algorithm.

Further data analysis showed us that in both PK and CO groups, we isolated lipophilic and non-lipophilic molecules with different molecular type ratios (data not shown). However, the best conditions were observed when all groups were aligned within one analysis pipeline. Within the aligned and identified annotated features, we observed that the molecules in the collagenase I group were at higher concentrations (Fig. 3A), indicating the higher efficiency of small molecule extraction for this method.

Enrichment analysis using identified small molecules showed more modulated pathways in these samples (Fig. 3C, Additional file 1: Table S2). The observed modulation of de novo fatty acid biosynthesis, Glycosphingolipid metabolism, carnitine shuttle, and purine metabolism in aged bone samples unveils an intricate web of molecular interactions that collectively impact bone health during aging. De novo fatty acid biosynthesis provides the necessary substrates for synthesizing lipids, including glycosphingolipids, essential components of cell membranes. The crossroads between these pathways occur with the insertion of new fatty acids into the complex lipid structures, influencing membrane fluidity and cellular signaling, ultimately connecting de novo fatty acid biosynthesis with glycosphingolipid metabolism. Furthermore, the Carnitine shuttle is a key route in transporting fatty acids into mitochondria for oxidation, ensuring an energy supply for bone cells. The balance of these pathways is under the regulatory control of transcription factors like peroxisome proliferator-activated receptors (PPARs), which can modulate gene expression in both fatty acid synthesis and oxidation, thereby coordinating the cellular response to metabolic demands. This regulation of lipid metabolism in bone cells underpins the finely tuned balance between anabolism and catabolism. Alterations in these pathways can lead to imbalances, potentially contributing to age-related bone conditions.

Consequently, proteins and genes involved in these pathways emerge as promising candidates for understanding the molecular mechanisms underlying age-related bone conditions. For instance, Fatty Acid Synthase (FASN), a key enzyme in de novo fatty acid biosynthesis, can be a valuable biomarker candidate due to its central role in lipid synthesis. Likewise, acid sphingomyelinase (ASM or SMPD1), a key player in glycosphingolipid metabolism, could be considered a biomarker reflecting the status of these pathways. Transcription factors such as PPARs, which regulate the balance between fatty acid synthesis and oxidation, may indicate pathway dysregulation. Additionally, components of the carnitine shuttle and proteins involved in energy balance, like Acyl-CoA Oxidase (ACOX1), might provide insights into energy utilization in bone cells. These candidates can offer insights into the molecular underpinnings of age-related bone disorders, serving as diagnostic or prognostic tools for assessing bone health in aging individuals and, in turn, guiding potential preventive therapeutic strategies. Understanding these pathways and their related proteins and genes is paramount for unraveling the complex molecular mechanisms underlying bone aging, opening the door to potential interventions that can mitigate the effects of aging on bone cells and tissues.

## Conclusions

This work presented a novel optimized analytical method that helped better identify key metabolites in the aged bone tissue. High-quality metabolomics data containing low abundant molecules can lead to defining high-resolution metabolite signatures, which is necessary to conclude discoveries of biomarkers and/or pathological pathways. Our analysis showed the alterations in metabolic pathways in aging bone. PPARs, namely PPARγ, influence FASN activity, regulating lipid synthesis and affecting bone homeostasis. ASM, linked to sphingolipid metabolism, impacts cell signaling pathways involved in bone health. ACOX1, responsible for fatty acid oxidation, contributes to lipid metabolism, affecting bone turnover. Dysregulation of these components can disrupt lipid balance, influencing osteoblast and osteoclast function, thus influencing bone quality and turnover during aging. The connections between these enzymes underscore potential therapeutic targets for age-related bone disorders. Further studies are worthy to defy clear molecular pathways.

The approach presented in this study helps to profile small molecules in the bone samples with more detail and higher resolution, which can provide critical information for the prevention/prognosis of bone aging. However, The authors do not exclude the limitation of the cohort in this study. It was due, in part, to severe exclusion criteria since several comorbidities may occur in the elderly population. Further clinical studies are suggestable considering comorbidities, gender and BMI status of the elderly population.

### Supplementary Information

**Additional file 1: Fig. S1.** The SANIST AI-QC pipeline.** Fig. S2.** Fellowchart presentation of metabolite extraction in different experimental groups. Internal controls were added before the addition of first solvent for each group.** Fig. S3.** Data validation examples with locally constructed library in NIST software platform. The query molecules are in red, and the best match found in the library is in blue.** Table S1.** Functional Enrichment analysis for the modified metabolic pathways according to the identified metabolites according to similarity with HDMB.** Table S2.** Enrichment analysis for the pool of identified molecules’ chemical types.** Table S3.** Statistical comparison of cortical and trabecular bone metabolites extracted with different methods.

## Data Availability

Other than the supplementary materials. The raw data for this work is available at: 10.5281/zenodo.10058232.

## Abbreviations

ACOX1: Acyl-CoA Oxidase
ASM: Acid sphingomyelinase
BRUs: Bone remodeling units
CO: Collagenase I
DC: Decalcification
EDTA: Ethylenediaminetetraacetic acid
FASN: Fatty Acid Synthase
HMDB: Human Metabolome database
HRMS: High-resolution mass spectrometry
MeOH: Methanol
MO: Matyash
MTBE: Methyl tert-butyl ether
MTM: Modified Matyash
PK: Proteinase K
PPARs: Peroxisome proliferator-activated receptors
