# Correction: Enhanced molecular release from elderly bone samples using collagenase I: insights into fatty acid metabolism alterations

**DOI:** 10.1186/s12967-024-05074-1

**Published:** 2024-03-24

**Authors:** Amir Mohammad Malvandi, Esra Halilaj, Martina Faraldi, Laura Mangiavini, Simone Cristoni, Valerio Leoni, Giovanni Lombardi

**Affiliations:** 1 Laboratory of Experimental Biochemistry & Molecular Biology, IRCCS Istituto Ortopedico Galeazzi, Via Cristina Belgioioso 173, 20157 Milan, Italy; 2 School of Biosciences and Veterinary Medicine, University of Camerino, Camerino, Italy; 3 Department of Biomedical Sciences for Health, University of Milan, Milan, Italy; 4 ISB-Ion Source & Biotechnologies Srl, Bresso, Italy; 5 Department of Laboratory Medicine, University of Milano-Bicocca, Azienda Socio Sanitaria Territoriale Della Brianza, ASST-Brianza, Desio Hospital, Desio, Italy; 6 Department of Athletics, Strength and Conditioning, Poznań University of Physical Education, Poznań, Poland

**Correction : Journal of Translational Medicine (2024) 22:143** 10.1186/s12967-024-04948-8

Following publication of the original article [1], we have been notified that affiliation 1 was not mentioned correctly.

It is now:

- 1 Laboratory of Experimental Biochemistry & Molecular Biology, IRCCS Istituto Ortopedico Galeazzi—Sant’Ambrogio, Via Cristina Belgioioso 173, 20157 Milan, Italy.

It should be as per below:

- 1 Laboratory of Experimental Biochemistry & Molecular Biology, IRCCS Istituto Ortopedico Galeazzi, Via Cristina Belgioioso 173, 20157 Milan, Italy.

The original article was updated.
