# Supplementary material for: Enhanced molecular release from elderly bone samples using collagenase I: insights into fatty acid metabolism alterations
Source: J Transl Med. 2024 Feb 9;22:143. doi: 10.1186/s12967-024-04948-8 (PMC10858523; doi:10.1186/s12967-024-04948-8)
Supplement: Supplementary file 1 — Additional file 1: Fig. S1. The SANIST AI-QC pipeline. Fig. S2. Fellowchart presentation of metabolite extraction in different experimental groups. Internal controls were added before the addition of first solvent for each group. Fig. S3. Data validation examples with locally constructed library in NIST software platform. The query molecules are in red, and the best match found in the library is in blue. Table S1. Functional Enrichment analysis for the modified metabolic pathways according to the identified metabolites according to similarity with HDMB. Table S2. Enrichment analysis for the pool of identified molecules’ chemical types. Table S3. Statistical comparison of cortical and trabecular bone metabolites extracted with different methods. [file 12967_2024_4948_MOESM1_ESM.docx]

*Supplementary materials*

**Enhanced molecular release from elderly bone samples using collagenase I: insights into fatty acid metabolism alterations**

Amir Mohammad Malvandi, Esra Halilaj, Martina Faraldi, Laura Mangiavini, Simone Cristoni, Valerio Leoni, Giovanni Lombardi


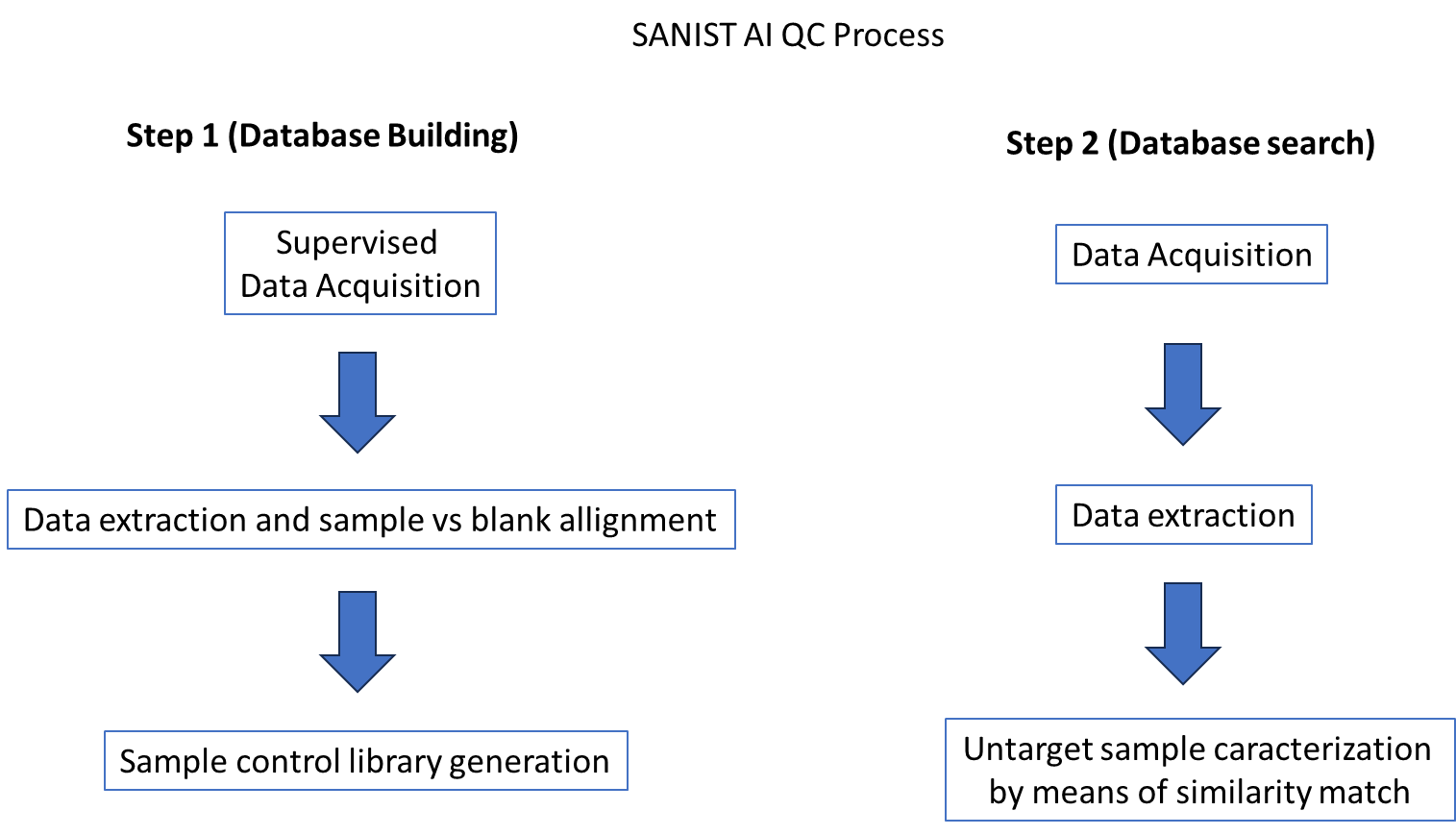


Fig. S1. The SANIST AI-QC pipeline.


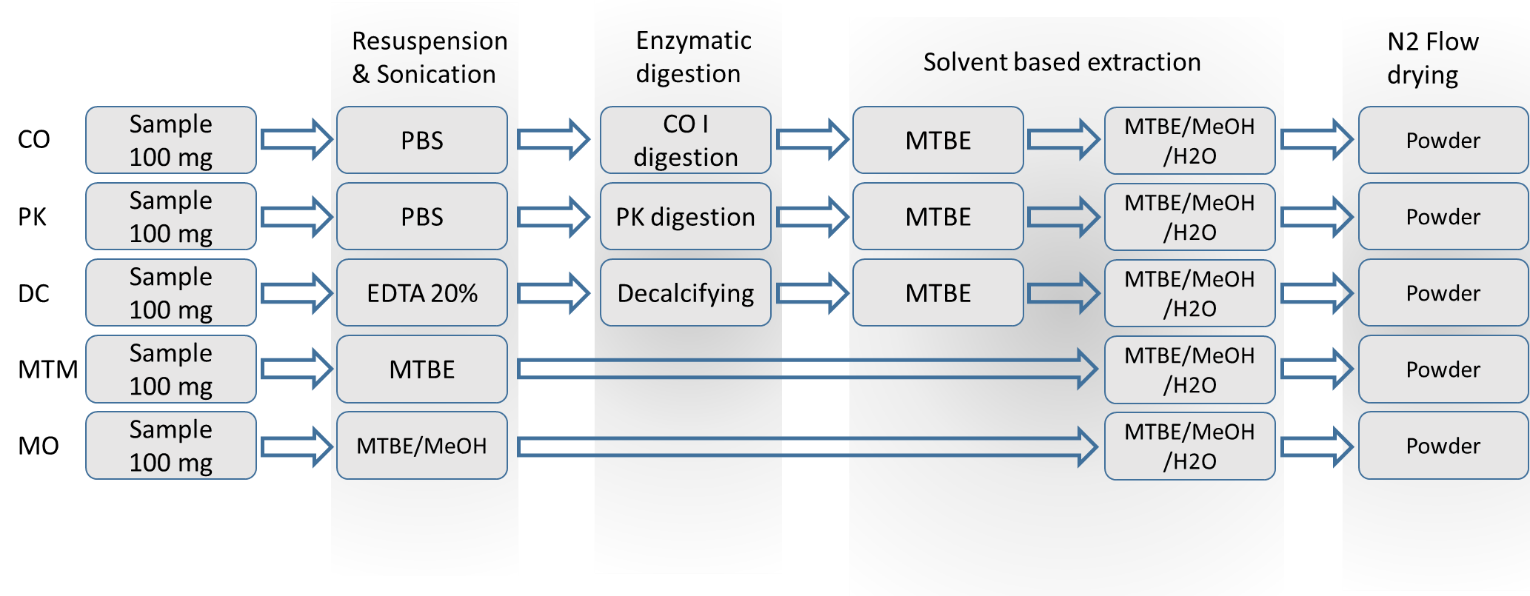


Fig. S2. Fellowchart presentation of metabolite extraction in different experimental groups. Internal controls were added before the addition of first solvent for each group.

Fig. S3. Data validation examples with locally constructed library in NIST software platform. The query molecules are in red, and the best match found in the library is in blue.

Table S1. Functional Enrichment analysis for the modified metabolic pathways according to the identified metabolites according to similarity with HDMB.

|  | Pathway total | Hits.total | Hits.sig | Expected | P(Fisher) | P(EASE) | P(Gamma) | Emp.Hits | Empirical | AdjP.Fisher | AdjP.EASE | AdjP.Gamma | Pathway Number | cpd.hits |
| --- | --- | --- | --- | --- | --- | --- | --- | --- | --- | --- | --- | --- | --- | --- |
| De novo fatty acid biosynthesis | 106 | 6 | 3 | 0.53892 | 0.006215 | 0.063098 | 0.000591 | 0 | 0 | 0.186453 | 1 | 0.017724 | P1 | EC00010;EC000154;EC0007 |
| Glycosphingolipid metabolism | 67 | 3 | 2 | 0.32335 | 0.023627 | 0.24396 | 0.000637 | 0 | 0 | 0.685183 | 1 | 0.018471 | P2 | EC0007;EC000118 |
| Carnitine shuttle | 72 | 5 | 2 | 0.43114 | 0.044611 | 0.31196 | 0.000698 | 0 | 0 | 1 | 1 | 0.019531 | P3 | EC000188;EC000154 |
| Purine metabolism | 80 | 4 | 2 | 0.43114 | 0.044611 | 0.31196 | 0.000698 | 0 | 0 | 1 | 1 | 0.019531 | P4 | EC0007;EC00084 |
| Methionine and cysteine metabolism | 94 | 5 | 2 | 0.53892 | 0.070209 | 0.37418 | 0.00078 | 0 | 0 | 1 | 1 | 0.020271 | P5 | EC0007;EC000102 |
| Propanoate metabolism | 31 | 1 | 1 | 0.10778 | 0.09375 | 1 | 0.000864 | 0 | 0 | 1 | 1 | 0.021601 | P6 | EC00010 |
| Fatty acid oxidation | 35 | 1 | 1 | 0.10778 | 0.09375 | 1 | 0.000864 | 0 | 0 | 1 | 1 | 0.021601 | P7 | EC000154 |
| Fatty acid oxidation, peroxisome | 28 | 1 | 1 | 0.10778 | 0.09375 | 1 | 0.000864 | 0 | 0 | 1 | 1 | 0.021601 | P8 | EC000154 |
| Keratan sulfate biosynthesis | 66 | 1 | 1 | 0.10778 | 0.09375 | 1 | 0.000864 | 0 | 0 | 1 | 1 | 0.021601 | P9 | EC0007 |
| Proteoglycan biosynthesis | 27 | 1 | 1 | 0.10778 | 0.09375 | 1 | 0.000864 | 0 | 0 | 1 | 1 | 0.021601 | P10 | EC0007 |
| Leukotriene metabolism | 92 | 13 | 3 | 1.509 | 0.13041 | 0.35757 | 0.001015 | 19 | 0.19 | 1 | 1 | 0.021601 | P11 | EC00042;EC000180;EC000150 |
| Tryptophan metabolism | 94 | 7 | 3 | 0.75449 | 0.13156 | 0.48307 | 0.00102 | 4 | 0.04 | 1 | 1 | 0.021601 | P12 | EC0009;EC00082 |
| Hexose phosphorylation | 20 | 3 | 2 | 0.21557 | 0.17916 | 1 | 0.00126 | 0 | 0 | 1 | 1 | 0.022671 | P13 | EC00011 |
| Xenobiotics metabolism | 110 | 2 | 1 | 0.21557 | 0.17916 | 1 | 0.00126 | 0 | 0 | 1 | 1 | 0.022671 | P14 | EC0007 |
| Phosphatidylinositol phosphate metabolism | 59 | 2 | 1 | 0.21557 | 0.17916 | 1 | 0.00126 | 20 | 0.2 | 1 | 1 | 0.022671 | P15 | EC00011 |
| Starch and Sucrose Metabolism | 33 | 3 | 2 | 0.21557 | 0.17916 | 1 | 0.00126 | 11 | 0.11 | 1 | 1 | 0.022671 | P16 | EC00011 |
| Tyrosine metabolism | 160 | 20 | 3 | 2.0479 | 0.2563 | 0.52134 | 0.001781 | 1 | 0.01 | 1 | 1 | 0.02493 | P17 | EC00082;EC00057;EC0007 |
| Glycosphingolipid biosynthesis - ganglioseries | 62 | 2 | 1 | 0.32335 | 0.25692 | 1 | 0.001786 | 0 | 0 | 1 | 1 | 0.02493 | P18 | EC0007 |
| Omega-3 fatty acid metabolism | 39 | 4 | 2 | 0.32335 | 0.25692 | 1 | 0.001786 | 14 | 0.14 | 1 | 1 | 0.02493 | P19 | EC000154 |
| Fatty acid activation | 74 | 4 | 1 | 0.32335 | 0.25692 | 1 | 0.001786 | 7 | 0.07 | 1 | 1 | 0.02493 | P20 | EC000154 |
| Pyrimidine metabolism | 70 | 3 | 1 | 0.32335 | 0.25692 | 1 | 0.001786 | 0 | 0 | 1 | 1 | 0.02493 | P21 | EC0007 |
| Omega-6 fatty acid metabolism | 55 | 3 | 1 | 0.32335 | 0.25692 | 1 | 0.001786 | 12 | 0.12 | 1 | 1 | 0.02493 | P22 | EC000154 |
| Fatty Acid Metabolism | 63 | 5 | 1 | 0.43114 | 0.32769 | 1 | 0.002468 | 5 | 0.05 | 1 | 1 | 0.02493 | P23 | EC00010 |
| Arachidonic acid metabolism | 95 | 10 | 1 | 0.43114 | 0.32769 | 1 | 0.002468 | 69 | 0.69 | 1 | 1 | 0.02493 | P24 | EC00042 |
| Sialic acid metabolism | 107 | 4 | 1 | 0.53892 | 0.39206 | 1 | 0.003333 | 27 | 0.27 | 1 | 1 | 0.02493 | P25 | EC00011 |
| Galactose metabolism | 41 | 8 | 4 | 0.53892 | 0.39206 | 1 | 0.003333 | 56 | 0.56 | 1 | 1 | 0.02493 | P26 | EC00011 |
| Linoleate metabolism | 46 | 8 | 1 | 0.64671 | 0.45058 | 1 | 0.004405 | 45 | 0.45 | 1 | 1 | 0.02493 | P27 | EC000182 |
| Vitamin A (retinol) metabolism | 67 | 9 | 1 | 0.75449 | 0.50375 | 1 | 0.005708 | 29 | 0.29 | 1 | 1 | 0.02493 | P28 | EC000167 |
| C21-steroid hormone biosynthesis and metabolism | 112 | 24 | 2 | 2.0479 | 0.55447 | 0.84344 | 0.007354 | 64 | 0.64 | 1 | 1 | 0.02493 | P29 | EC000192;EC0007 |
| Androgen and estrogen biosynthesis and metabolism | 95 | 24 | 1 | 1.4012 | 0.73387 | 1 | 0.019356 | 84 | 0.84 | 1 | 1 | 0.02493 | P30 | EC0007 |

Table S2. Enrichment analysis for the pool of identified molecules’ chemical types.

|  | total | expected | hits | Raw p | Holm p | FDR |
| --- | --- | --- | --- | --- | --- | --- |
| Amino acids | 277 | 0.145 | 13 | 6.34E-22 | 4.63E-19 | 4.63E-19 |
| C19 steroids | 98 | 0.0514 | 7 | 1.18E-13 | 8.63E-11 | 4.32E-11 |
| Porphyrins | 6 | 0.00315 | 4 | 1.07E-12 | 7.80E-10 | 2.61E-10 |
| C21 steroids | 148 | 0.0776 | 6 | 2.23E-10 | 1.62E-07 | 4.07E-08 |
| Leukotrienes | 69 | 0.0362 | 4 | 6.01E-08 | 4.37E-05 | 8.79E-06 |
| Acyl CoAs | 196 | 0.103 | 5 | 7.61E-08 | 5.53E-05 | 9.28E-06 |
| Glucuronides | 52 | 0.0273 | 3 | 3.04E-06 | 0.0022 | 0.000317 |
| HETE | 60 | 0.0315 | 3 | 4.69E-06 | 0.0034 | 0.000429 |
| Fatty acyl thioesters | 222 | 0.116 | 4 | 6.44E-06 | 0.00466 | 0.000523 |
| Epoxyeicosatrienoic acids | 8 | 0.0042 | 2 | 7.61E-06 | 0.0055 | 0.000556 |
| Disaccharides | 9 | 0.00472 | 2 | 9.78E-06 | 0.00705 | 0.00065 |
| Glycosylamines | 10 | 0.00524 | 2 | 1.22E-05 | 0.0088 | 0.000745 |
| Methoxyphenols | 19 | 0.00997 | 2 | 4.63E-05 | 0.0333 | 0.0026 |
| Monosaccharide phosphates | 24 | 0.0126 | 2 | 7.46E-05 | 0.0536 | 0.0039 |
| Hydroxysteroids | 30 | 0.0157 | 2 | 0.000117 | 0.0842 | 0.00572 |
| Organooxygen compounds | 31 | 0.0163 | 2 | 0.000125 | 0.0898 | 0.00573 |
| Androstane steroids | 35 | 0.0184 | 2 | 0.00016 | 0.115 | 0.00683 |
| Prostaglandins | 199 | 0.104 | 3 | 0.000168 | 0.12 | 0.00683 |
| Pyrimidine ribonucleoside triphosphates | 2 | 0.00105 | 1 | 0.00105 | 0.748 | 0.0403 |
| Pyrimidine deoxyribonucleoside triphosphates | 3 | 0.00157 | 1 | 0.00157 | 1 | 0.0575 |
| Phenylpyruvic acid derivatives | 4 | 0.0021 | 1 | 0.0021 | 1 | 0.0697 |
| N-arylamides | 4 | 0.0021 | 1 | 0.0021 | 1 | 0.0697 |
| Phenylpropylamines | 5 | 0.00262 | 1 | 0.00262 | 1 | 0.0833 |
| Short-chain acids and derivatives | 7 | 0.00367 | 1 | 0.00367 | 1 | 0.107 |
| Acetamides | 7 | 0.00367 | 1 | 0.00367 | 1 | 0.107 |
| Purine ribonucleoside diphosphates | 8 | 0.0042 | 1 | 0.00419 | 1 | 0.118 |
| Phenethylamines | 9 | 0.00472 | 1 | 0.00471 | 1 | 0.123 |
| Morphinans | 9 | 0.00472 | 1 | 0.00471 | 1 | 0.123 |
| Catechols | 11 | 0.00577 | 1 | 0.00575 | 1 | 0.145 |
| Hippuric acids | 1470 | 0.77 | 4 | 0.00766 | 1 | 0.185 |
| Phenylacetic acids | 15 | 0.00787 | 1 | 0.00784 | 1 | 0.185 |
| Hydroxycinnamic acids | 16 | 0.00839 | 1 | 0.00836 | 1 | 0.191 |
| Unsaturated Fatty Acids | 267 | 0.14 | 2 | 0.00884 | 1 | 0.191 |
| Benzoic acid esters | 17 | 0.00892 | 1 | 0.00888 | 1 | 0.191 |
| Carbohydrates and carbohydrate conjugates | 22 | 0.0115 | 1 | 0.0115 | 1 | 0.24 |
| Amino Fatty Acids | 23 | 0.0121 | 1 | 0.012 | 1 | 0.244 |
| Ubiquinones | 27 | 0.0142 | 1 | 0.0141 | 1 | 0.278 |
| Polyprenols | 34 | 0.0178 | 1 | 0.0177 | 1 | 0.34 |
| Fatty aldehydes | 52 | 0.0273 | 1 | 0.0269 | 1 | 0.504 |
| Triterpenoids | 511 | 0.268 | 2 | 0.0299 | 1 | 0.546 |
| Retinoids | 70 | 0.0367 | 1 | 0.0361 | 1 | 0.643 |
| C27 bile acids | 113 | 0.0593 | 1 | 0.0576 | 1 | 1 |
| Glycerophosphoinositols | 125 | 0.0656 | 1 | 0.0635 | 1 | 1 |
| C24 bile acids | 150 | 0.0787 | 1 | 0.0757 | 1 | 1 |
| C30 isoprenoids | 174 | 0.0913 | 1 | 0.0873 | 1 | 1 |
| Fatty acyl CoAs | 178 | 0.0934 | 1 | 0.0892 | 1 | 1 |
| Cholesterol and derivatives | 203 | 0.106 | 1 | 0.101 | 1 | 1 |
| C10 isoprenoids | 242 | 0.127 | 1 | 0.119 | 1 | 1 |
| C20 isoprenoids | 281 | 0.147 | 1 | 0.137 | 1 | 1 |
| Glycosphingolipids | 452 | 0.237 | 1 | 0.211 | 1 | 1 |
| Sesquiterpenoids | 452 | 0.237 | 1 | 0.211 | 1 | 1 |
| Triradylcglycerols | 38800 | 20.4 | 1 | 1 | 1 | 1 |

**Table S3. Statistical comparison of cortical and trabecular bone metabolites extracted with different methods.**

The Number of extracted metabolites is expressed as absolute values. Chi-Square test on R software version 4.3.0 was used to compare cortical and trabecular bone samples’ metabolites extracted by MO, MTM, DC, PK, CO methods. Table A shows the comparison between all methods. Table B shows the comparisons between every two methods used in this study.

| A | ***MO*** | ***MTM*** | ***DC*** | ***PK*** | ***CO*** | ***X*² Test**  **df()** | **p-Value** |
| --- | --- | --- | --- | --- | --- | --- | --- |
| ***Cortical bone*** | 1186 | 1402 | 1757 | 2117 | 2708 | 794.12 (4) | < 0,0001 |
| ***Trabecular bone*** | 1204 | 1286 | 1439 | 1922 | 2153 | 431.55 (4) | < 0,0001 |

| B | **Cortical bone** | ***X*² Test df()** | **p-Value** | **Trabecular bone** | ***X*² Test df()** | **p-Value** |
| --- | --- | --- | --- | --- | --- | --- |
| **MO** | 1186 | 18.03 (1) | < 0.0001 | 1204 | 2.70 (1) | 0.100 |
| **MTM** | 1402 |  |  | 1286 |  |  |
| **MO** | 1186 | 110.79(1) | < 0.0001 | 1204 | 20.89 (1) | < 0.0001 |
| **DC** | 1757 |  |  | 1439 |  |  |
| **MO** | 1186 | 262.42 (1) | < 0.0001 | 1204 | 164.91 (1) | < 0.0001 |
| **PK** | 2117 |  |  | 1922 |  |  |
| **MO** | 1186 | 594.89 (1) | < 0.0001 | 1204 | 268.28 (1) | < 0.0001 |
| **CO** | 2708 |  |  | 2153 |  |  |
| **MTM** | 1402 | 39.89 (1) | < 0.0001 | 1286 | 8.59 (1) | 0.003 |
| **DC** | 1757 |  |  | 1439 |  |  |
| **MTM** | 1402 | 145.28 (1) | < 0.0001 | 1286 | 126.09 (1) | < 0.0001 |
| **PK** | 2117 |  |  | 1922 |  |  |
| **MTM** | 1402 | 415(1) | < 0.0001 | 1286 | 218.58 (1) | < 0.0001 |
| **CO** | 2708 |  |  | 2153 |  |  |
| **DC** | 1757 | 33.45(1) | < 0.0001 | 1439 | 69.41 (1) | < 0.0001 |
| **PK** | 2117 |  |  | 1922 |  |  |
| **DC** | 1757 | 202.55 (1) | < 0.0001 | 1439 | 141.93 (1) | < 0.0001 |
| **CO** | 2708 |  |  | 2153 |  |  |
| **PK** | 2117 | 72.39(1) | < 0.0001 | 1922 | 13.09 (1) | < 0.0001 |
| **CO** | 2708 |  |  | 2153 |  |  |
